# A biomechanical investigation of mandibular molar implants: reproducibility and validity of a finite element analysis model

**DOI:** 10.1186/s40729-015-0011-5

**Published:** 2015-04-28

**Authors:** Miyuki Omori, Yuji Sato, Noboru Kitagawa, Yuta Shimura, Manabu Ito

**Affiliations:** Department of Geriatric Dentistry, Showa University, School of Dentistry, 2-1-1 Kitasenzoku, Ota-ku, Tokyo, 145-8515 Japan

**Keywords:** Three-dimensional finite element analysis, FEA, Displacement, Stress distribution, Reproducibility, Validity

## Abstract

**Background:**

Three-dimensional finite element analysis (FEA) is effective in analyzing stress distributions around dental implants. However, FEA of living tissue involves many conditions, and the structures and behaviors are complex; thus, it is difficult to ensure the validity of the results. To verify reproducibility and validity, we embedded implants in experimental models and constructed FEA models; implant displacements were compared under various loading conditions.

**Methods:**

Implants were embedded in the molar regions of artificial mandibles to fabricate three experimental models. A titanium superstructure was fabricated and three loading points (buccal, central, and lingual) were placed on a first molar. A vertical load of 100 N was applied to each loading point and implant displacements were measured. Next, the experimental models were scanned on micro-computed tomography (CT) and three-dimensional FEA software was used to construct two model types. A model where a contact condition was assumed for the implant and artificial mandible (a contact model) was constructed, as was a model where a fixation condition was assumed (a fixation model). The FEA models were analyzed under similar conditions as the experimental models; implant displacements under loading conditions were compared between the experimental and FEA models. Reproducibility of the models was assessed using the coefficient of variation (CV), and validity was assessed using a correlation coefficient.

**Results:**

The CV of implant displacement was 5% to 10% in the experimental and FEA models under loading conditions. Absolute values of implant displacement under loading were smaller in FEA models than the experimental model, but the displacement tendency at each loading site was similar. The correlation coefficient between the experimental and contact models for implant displacement under loading was 0.925 (*p* < 0.01). The CVs of equivalent stress values in the FEA models were 0.52% to 45.99%.

**Conclusions:**

Three-dimensional FEA models were reflective of experimental model displacements and produced highly valid results. Three-dimensional FEA is effective for investigating the behavioral tendencies of implants under loading conditions. However, the validity of the absolute values was low and the reproducibility of the equivalent stresses was inferior; thus, the results should be interpreted with caution.

## Background

Bone remodeling to maintain osseointegration between bone and implant is absolutely essential to ensure favorable results and long-term stability in implant treatment [1,2]. Bone remodeling requires that various stresses generated around the bone caused by the occlusal load applied to the implant be within an appropriate range. Concentrations of stress at the bone-implant interface, which are caused by overloading, have been reported to result in bone resorption [3-5]. However, much remains to be understood about the relationship between mechanical stimulation of the bone and bone dynamics. It is therefore very important to shed light on how peri-implant bone is affected under various conditions, such as the positioning of the implant, placement angle, and bone quality. In recent years, a number of studies using biomechanical investigations have been performed to explore these clinical issues [6-10]. Photoelastic tests, strain gauge method, and three-dimensional finite element analyses (FEAs) have been used in typical biomechanical investigations. In experimental analyses, the photoelastic test and strain gauge method have the advantage of measuring the actual implant. However, the photoelastic test has the disadvantage that model fabrication is complicated. A disadvantage of the strain gauge method is that it is not possible to measure the subject’s entire stress. On the other hand, it is possible to use an FEA to ascertain the stress distributions of a subject’s interior, which are difficult to measure in an experimental analysis. To extract various physical data such as stress, strain, and displacement, conditions can be set more easily than in other biomechanical investigations [11]. This is the reason why FEAs have been studied in typical biomechanical investigations in recent years.

However, three-dimensional FEA of living tissue entails some disadvantages, including the large number of condition settings and assumptions often included and the complexity of internal structures and behaviors. Moreover, there are lingering questions about the reliability of results produced from a stress analysis, and it is difficult to ensure the validity of the results. One method to verify the validity of three-dimensional FEA models is to carry out experimental analyses in parallel to confirm the extent to which actual behaviors are reproduced and to determine the consistency in displacement between the two models [12-15]. In the future, it appears necessary to fabricate a three-dimensional FEA model that is reproducible and valid to continue revealing problems that arise when actual implant treatments are performed.

With the purpose of verifying the reproducibility and validity of a three-dimensional finite element model, the displacements of implants embedded in an experimental model and in three-dimensional FEA models constructed from the experimental model were compared under various loading conditions.

## Methods

### Fabrication of the experimental model

#### Artificial mandibular bone

**Figure 1:**
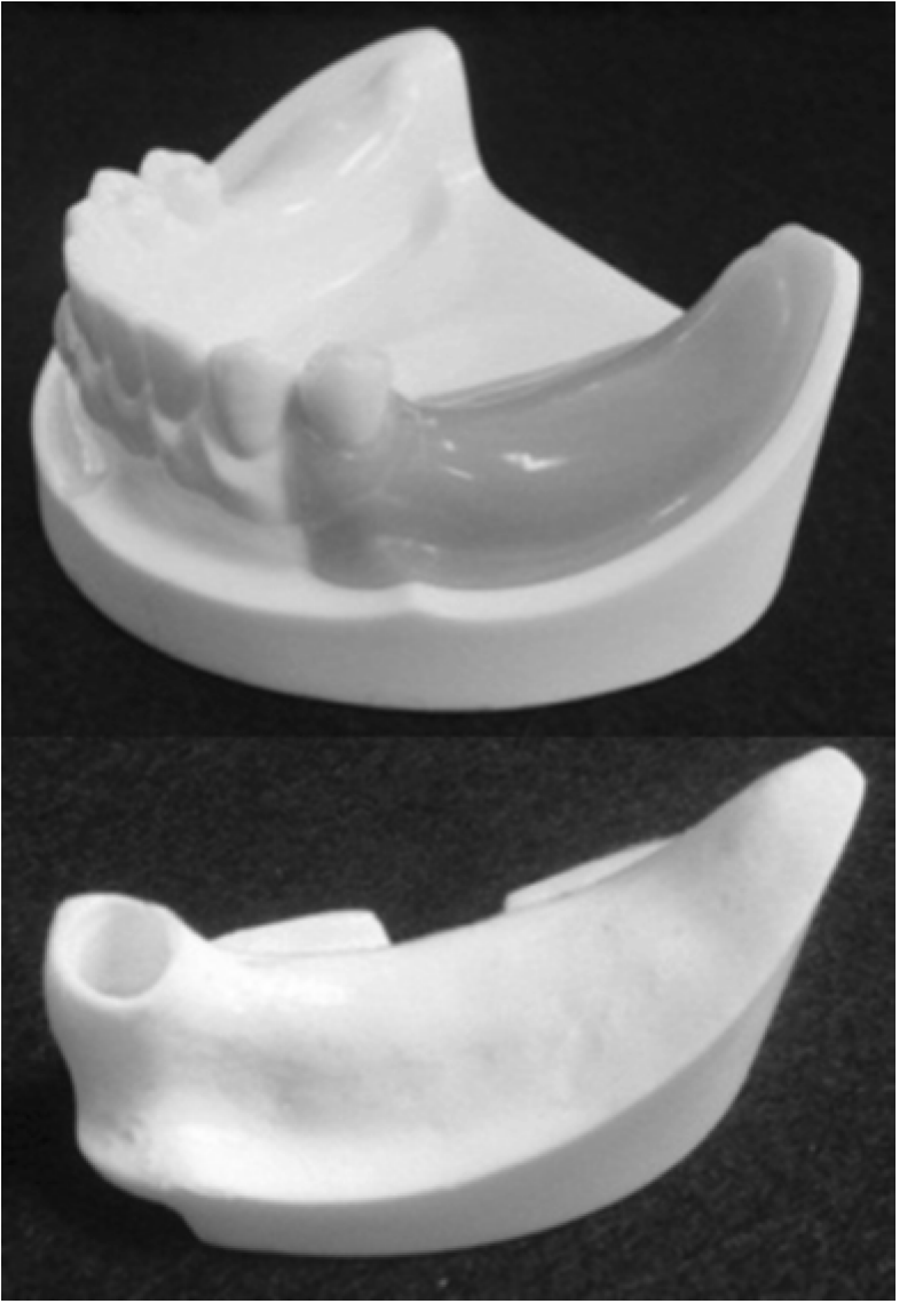
An artificial mandible.

An artificial mandibular bone (P9-X.1135, Nissin Dental Products, Kyoto, Japan) with free-end edentulism of the left mandibular first premolar (no. 34), second premolar (no. 35), and first molar (no. 36) was used (Figure 1). The model is composed of a two-layer structure of artificial cortical bone (urethane resin) and artificial cancellous bone (urethane resin foam).

#### Implant placement

Using the anatomical crown width diameter as a reference [16], we embedded three implants. The distance between the second premolar and mandibular first premolar implants was 8 mm. The distance between the first molar and second premolar implants was 10 mm.

**Figure 2:**
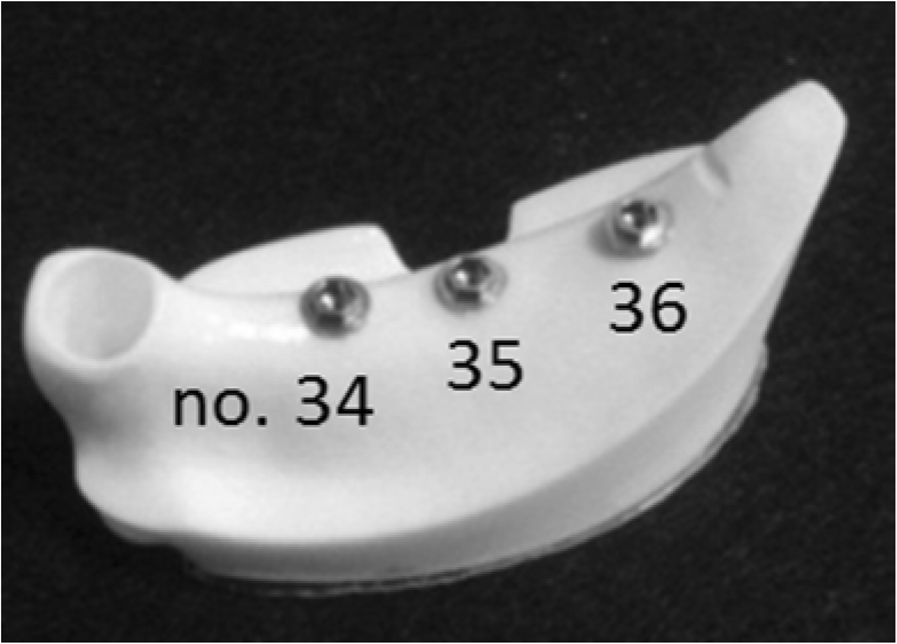
Three implants were embedded in an artificial mandible.

An implant placement guide (Landmark Guide™, iCAT, Osaka, Japan) was fabricated to precisely embed the implants in the artificial mandible. A drilling machine (Enkoh’s, Enshu Industrial, Shizuoka, Japan) and implant placement guide were used to embed the implants perpendicular to the bottom surface of the artificial mandible. A drill to form implant cavities (Brånemark System® Twist Drills, Nobel Biocare, Göteborg, Sweden) was mounted onto the drilling machine, and three implant cavities 3.0 mm in diameter and 10 mm in depth were formed. Then, in each of the implant cavities, an implant 3.75 mm in diameter and 10 mm in length (Brånemark System® Mk III, Nobel Biocare, Göteborg, Sweden) was embedded using 40 N · cm of torque (Figure 2).

#### Preparation of the superstructure

**Figure 3:**
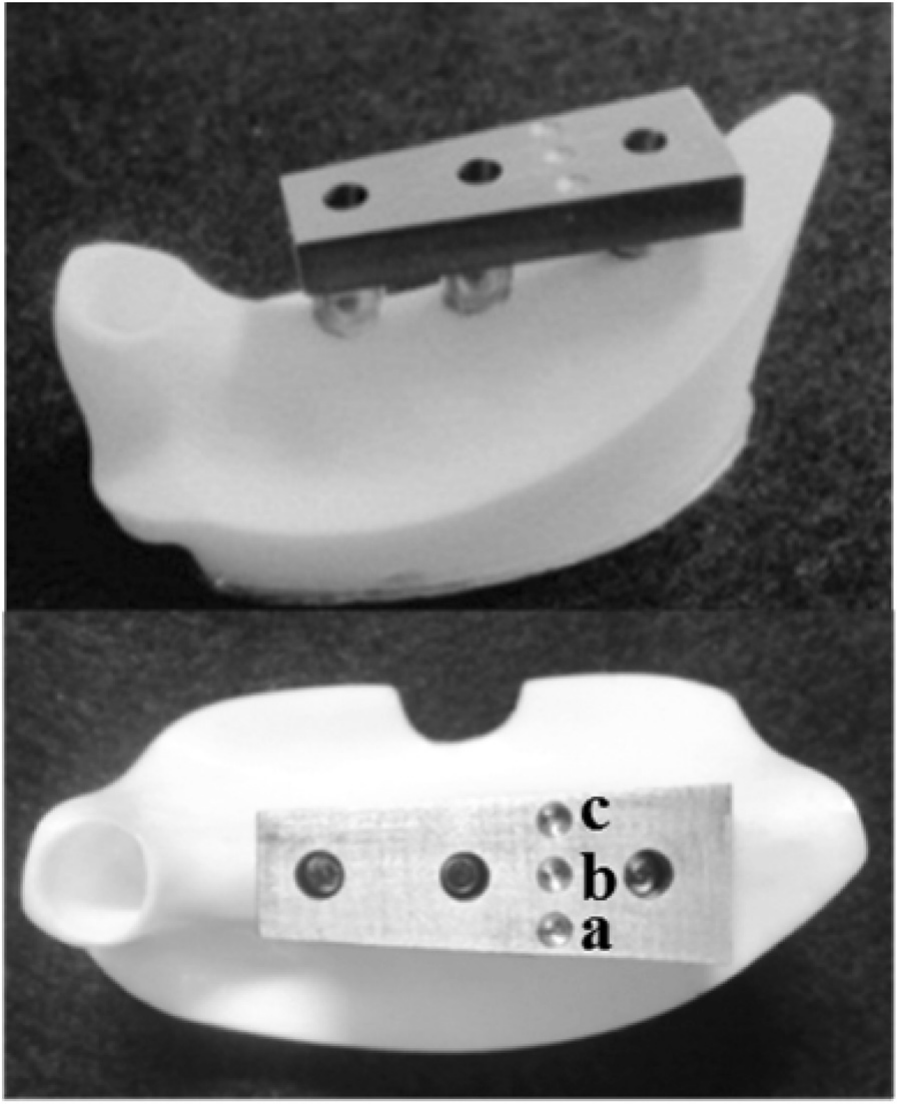
An experimental model. **(a)** Buccal loading, **(b)** central loading, and **(c)** lingual loading are shown.

Using the anatomical crown width as a reference [16], it was determined that the occlusal surface view of the superstructure would be trapezoidal with a 7-mm buccolingual width in the mesial first premolar section, a 10-mm buccolingual width in the distal first molar section, and a 26-mm mesiodistal width (Figure 3). The vertical dimension was 8 mm; the upper 4 mm was the thickness of the superstructure and the lower 4 mm was the abutment connection. Three loading points 2 mm in diameter and 0.2 mm in depth were applied to the occlusal surface of the first molar; these formed the buccal loading point (Figure 3a), central loading point (Figure 3b), and lingual loading point (Figure 3c). The superstructure was made of titanium (ISUS, DENTSPLY Sankin, Tokyo, Japan) and fabricated using computer-aided design/computer-aided manufacturing (CAD/CAM). Three experimental models were fabricated where the superstructure was mounted onto an implant-embedded artificial mandible.

### Construction of three-dimensional FEA models

The experimental models were fixed in a micro-CT scanner (inspeXio SMX-90CT, SHIMADZU, Kyoto, Japan) and scanned under the following imaging conditions: tube voltage, 90 kV; tube current, 109 nA; and slice thickness, 100 μm. FEA software (Mechanical Finder®, Research Center of Computational Mechanics, Tokyo, Japan) was used to construct three-dimensional FEA models from the resulting computed tomography (CT) data. The mesh was constructed of tetrahedral elements, and the total numbers of nodes and elements were approximately 270,000 and 1,500,000, respectively. For the Young’s modulus and Poisson ratio of each element, the artificial mandible manufacturer’s publicly disclosed values were used so that they would be similar to the physical properties of the experimental model. They were 628 MPa and 0.3 for artificial cancellous bone, 1,372 MPa and 0.3 for artificial cortical bone, and 100,800 MPa and 0.3 for the implant and superstructure (Table 1). The implant, abutment, and superstructure were assumed to be a continuous structure made of titanium; no intervening conditions were set between the implant and abutment, nor between the abutment and superstructure. The artificial cortical bone, artificial cancellous bone, implant, and superstructure were assumed to be homogeneous, isotropic, and linearly elastic.

**Table 1:** **Mechanical properties of the materials used in the FEA**

**Material**	**Young’s modulus (MPa)**	**Poisson ratio**
Artificial cancellous bone	628	0.3
Artificial cortical bone	1,373	0.3
Implant and superstructure	100,600	0.3

*FEA* finite element analysis.

To better understand how peri-implant bone is affected by differences in boundary conditions, two different kinds of models were fabricated by changing the boundary conditions between the implant and artificial mandibular bone. One was called a ‘contact model,’ in which the artificial mandible and implant were in complete contact. The coefficient of friction of the interface between the implants and artificial mandibular bones was set to zero. The boundary conditions of the experimental model were reproduced by the contact model of FEA. Immediate loading was assumed in this model, because a state of contact was reproduced between the implant and artificial mandibular bone. The other was called a ‘fixation model,’ in which the artificial mandible and implant were completely bonded together. Delayed loading after the acquisition of osseointegration was assumed in this model. Fixation models were constructed by changing the boundary conditions of the contact model.

### Displacement measurements

#### Implant displacement measurements under loading conditions in the experimental model

**Figure 4:**
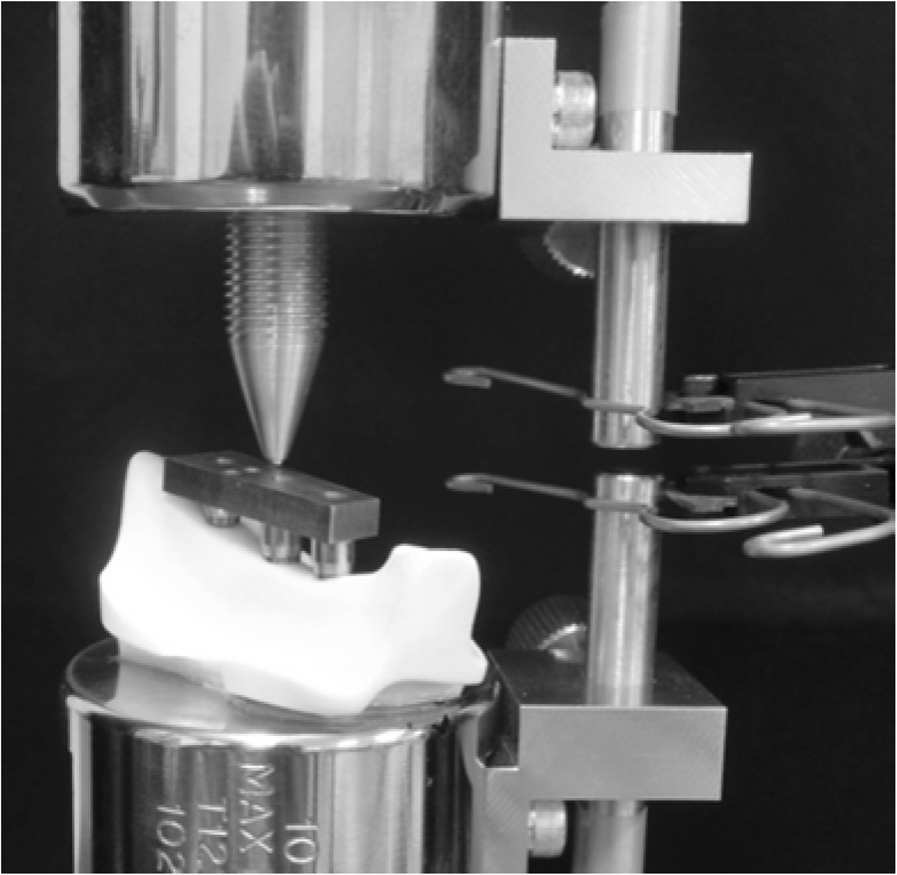
An experimental model loading test.

Implant displacement measurements under loading conditions were measured using an Instron-type universal testing machine (Instron‐5500R®, Instron Japan, Kanagawa, Japan) for the experimental model. The experimental models were placed on the worktable of an Instron-type universal testing machine, and compression tests were performed using a conical jig. A vertical load was applied at a rate of 0.5 mm/s to the three loading points. Using a report [17] stating that the maximum occlusal force applied to an implant superstructure in the molar region is 200 N as a reference, we selected 100 N for loading, assuming the forces used during mastication, which are not excessive occlusal forces. A strain gauge (2630-100, Instron Japan, Kanagawa, Japan) was attached between the worktable and jig, and the change in the distance between the worktable and jig was measured under the assumption that it would be the same as the implant displacements under loading conditions (Figure 4). Measurements were taken five times at each loading site, and the mean of the five measurements was taken as the representative value of the loading site in that model.

#### Implant displacement measurements under loading conditions in the FEA models

**Figure 5:**
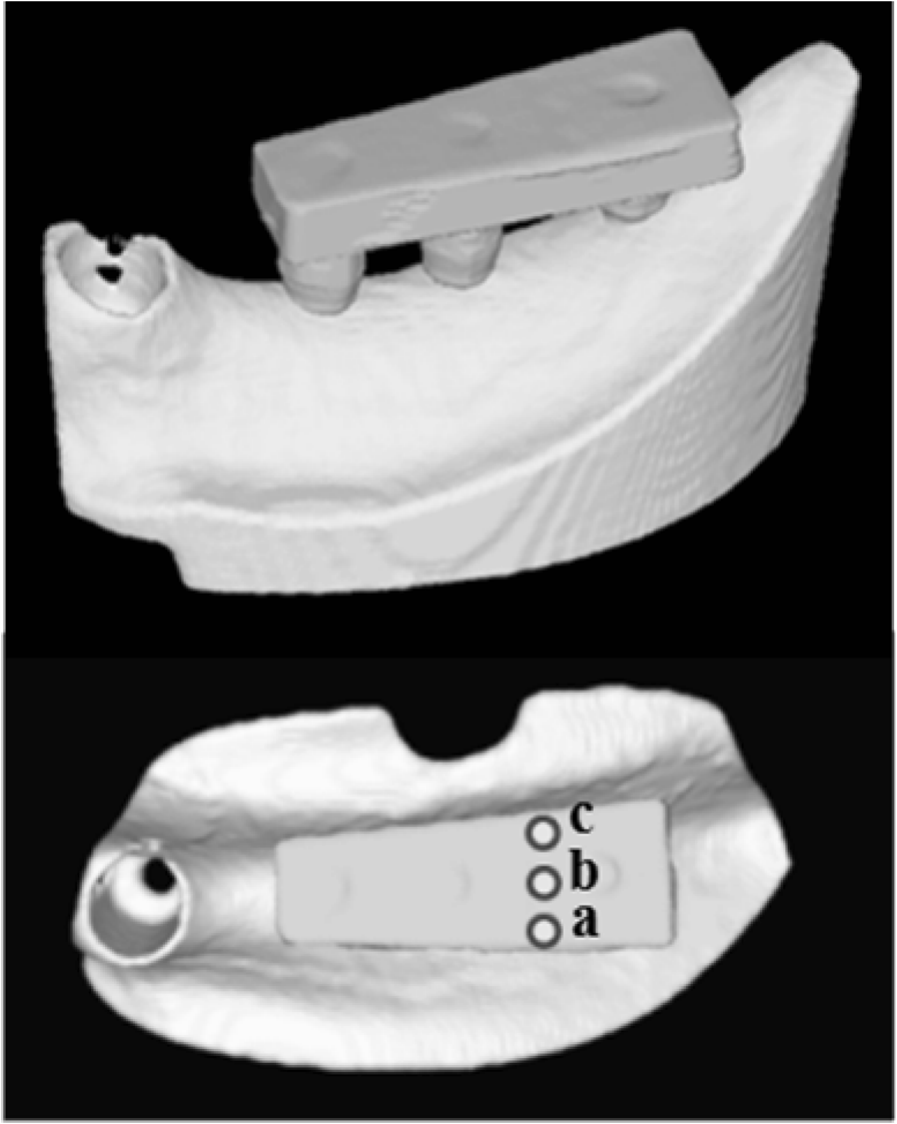
An FEA model. **(a)** Buccal loading, **(b)** central loading, and **(c)** lingual loading are shown.

All nodes at the bottom of the artificial mandible were completely restrained, 100 N of vertical load was applied to the three loading points, and an elastic analysis was performed. The vertical displacement of the loading points was assumed to be the displacement of the implants under loading conditions, and analyses were performed for the three loading sites (Figure 5).

#### Measurements of three-dimensional displacement in the FEA models

We analyzed the three-dimensional displacements of the three implants when 100 N of vertical load was applied. The assessment sites were the neck and tip of the implant, and the displacements of the implants under loading were analyzed with respect to the buccolingual direction (*x*-axis), the mesiodistal direction (*y*-axis), and the inferior-superior direction (*z*-axis).

### Assessments of stress distributions and values in the FEA models

The stress distribution (equivalent stress) generated in the interior of the artificial mandible under loading conditions was assessed. We also compared the equivalent stress values of each of loading point at the bone surrounding the necks and tips of the three implants.

### Statistical analysis

Regarding displacement under loading, a one-way analysis of variance (ANOVA) was used to investigate statistically significant differences between the loading sites. A three-way ANOVA was used to investigate statistically significant differences in three-dimensional implant displacements under loading conditions. The assessment site, dental formula, and loading point were used as intra-subject parameters.

Additionally, a three-way ANOVA was used to investigate statistically significant differences in equivalent stress values. The boundary conditions, dental formula, and loading point were used as intra-subject parameters.

To assess the reproducibility of each of the models, the coefficient of variation (CV) was calculated for implant displacement under loading conditions and the equivalent stress values from each of the three experimental and FEA models. To assess the validity of the FEA models, Pearson’s correlation coefficient was calculated for implant displacement under loading conditions in the experimental model and the contact model. Statistical processing was performed using PASW Statistics 18 (SPSS, Tokyo, Japan).

## Results

### Implant displacement under loading conditions

**Figure 6:**
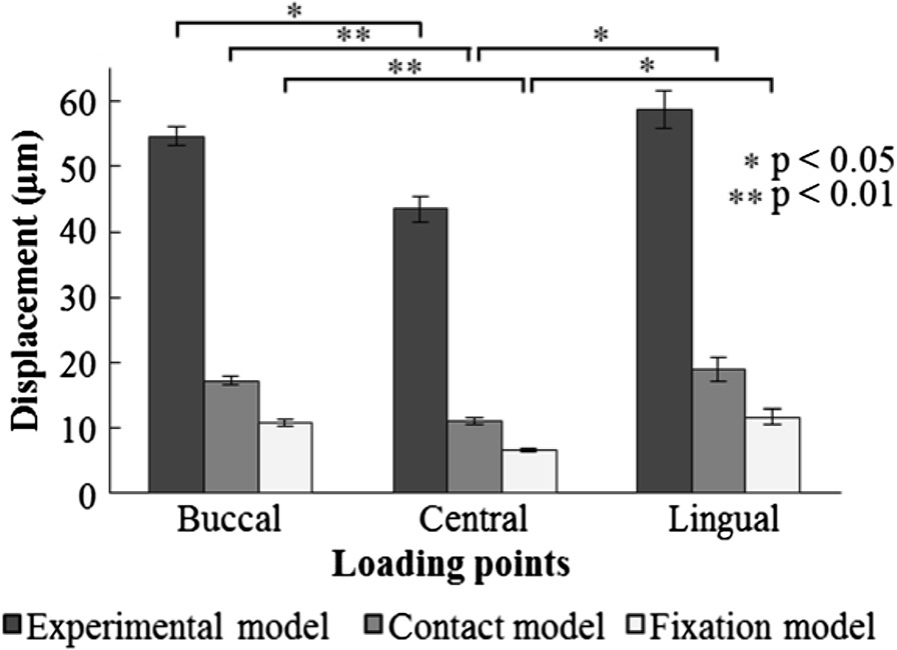
Implant displacement under loading conditions.

Figure 6 and Table 2 show the results for implant displacement under 100 N of vertical loading at each loading point and in each model.

**Table 2:** **Coefficients of variation in implant displacement under loading conditions**

**Model**	**Loading**			**Average**
	**Buccal loading**	**Central loading**	**Lingual loading**	
Experimental model	2.49	4.76	4.90	4.05
Contact model	4.55	4.48	9.64	6.22
Fixation model	5.26	4.85	9.26	6.45

Coefficient of variation (%) = (S.D.)/(mean).

The implant displacement under loading conditions in the experimental model and the two FEA models showed a tendency to exhibit the smallest values under central loading; substantially similar values were exhibited in buccal and lingual loading. Buccal loading (*p* < 0.05) in the experimental model and buccal (*p* < 0.01) and lingual loading (*p* < 0.05) values in the FEA models were significantly greater than the values obtained from central loading. The implant displacement under loading conditions in the FEA models showed lower values than in the experimental model at all loading points, but aspects of implant displacement under loading caused by differences in the loading point showed a similar tendency. The correlation coefficient between the experimental model and the contact model was 0.925, representing a significant and strong correlation (*p* < 0.01). The maximum CV value was 4.90% in the experimental model, 9.64% in the contact model, and 9.26% in the fixation model (Table 2).

### Three-dimensional displacements in the FEA models

**Figure 7:**
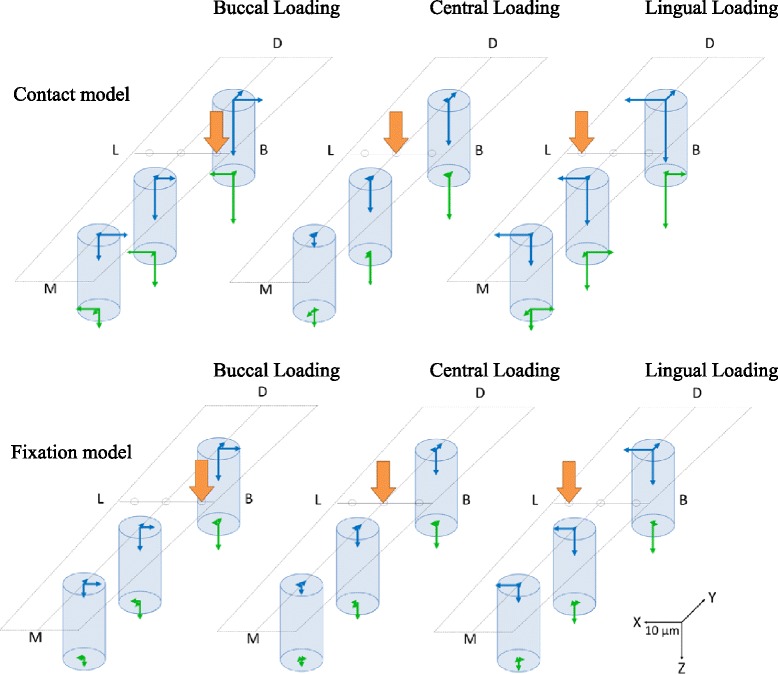
The displacement of the three implants. (M) Mesial side, (D) Distal side, (B) Buccal side, and (L) Lingual side are shown.

Figure 7 shows the results of three-dimensional implant displacement for each loading point under 100 N of vertical loading.

#### Three-dimensional displacement in the buccolingual direction (*x*-axis)

**Figure 8:**
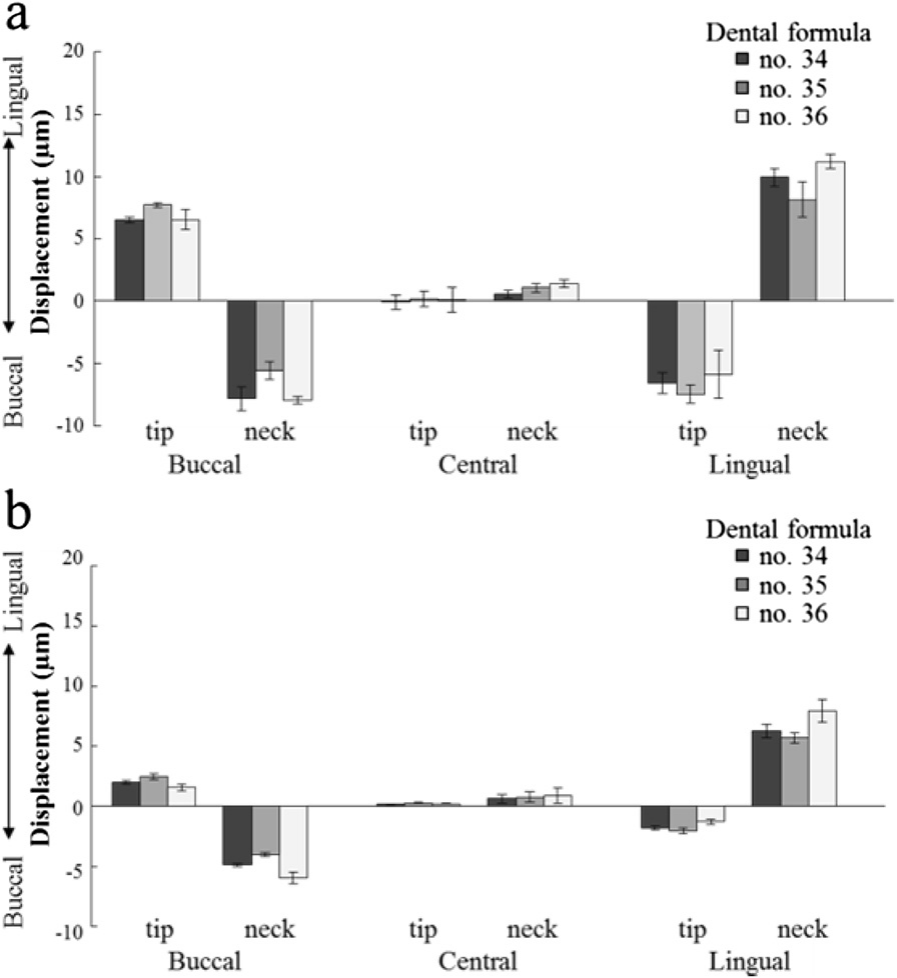
Displacement in the buccolingual direction (*x*-axis). **(a)** The contact model and **(b)** the fixation model.

Under buccal and lingual loading conditions, displacement involving rotation inclined towards the loaded side was exhibited; the displacements were substantially equal (Figure 8). Central loading resulted in the smallest displacement, and almost no displacement was observed. The fixation model had less displacement than the contact model. With regard to the aspects of displacement, similar tendencies were shown in both the contact model and the fixation model. The results of the ANOVA showed that for both the contact and fixation models, the loading site was a significant factor in the three-dimensional displacement (*p* < 0.01) (Table 3).

**Table 3:** **Three-way ANOVA (displacement in the buccolingual direction [** ***x*** **-axis])**

**Source**	**Sum of squares**	***df***	**Mean squared**	***F*** **value**	***p*** **value**
Contact model					
A: Observed area	16.346	1	16.346	4.362	0.172
B: Dental formula	2.106	2	1.053	5.019	0.081
C: Loading points	25.372	2	12.686	109.445	0.000**
Fixation model					
A: Observed area	5.568	1.000	5.568	9.006	0.095
B: Dental formula	0.294	2.000	0.147	5.323	0.075
C: Loading points	139.681	1.106	126.319	308.735	0.002**

**p < 0.01.

#### Three-dimensional displacement in the mesiodistal direction (*y*-axis)

**Figure 9:**
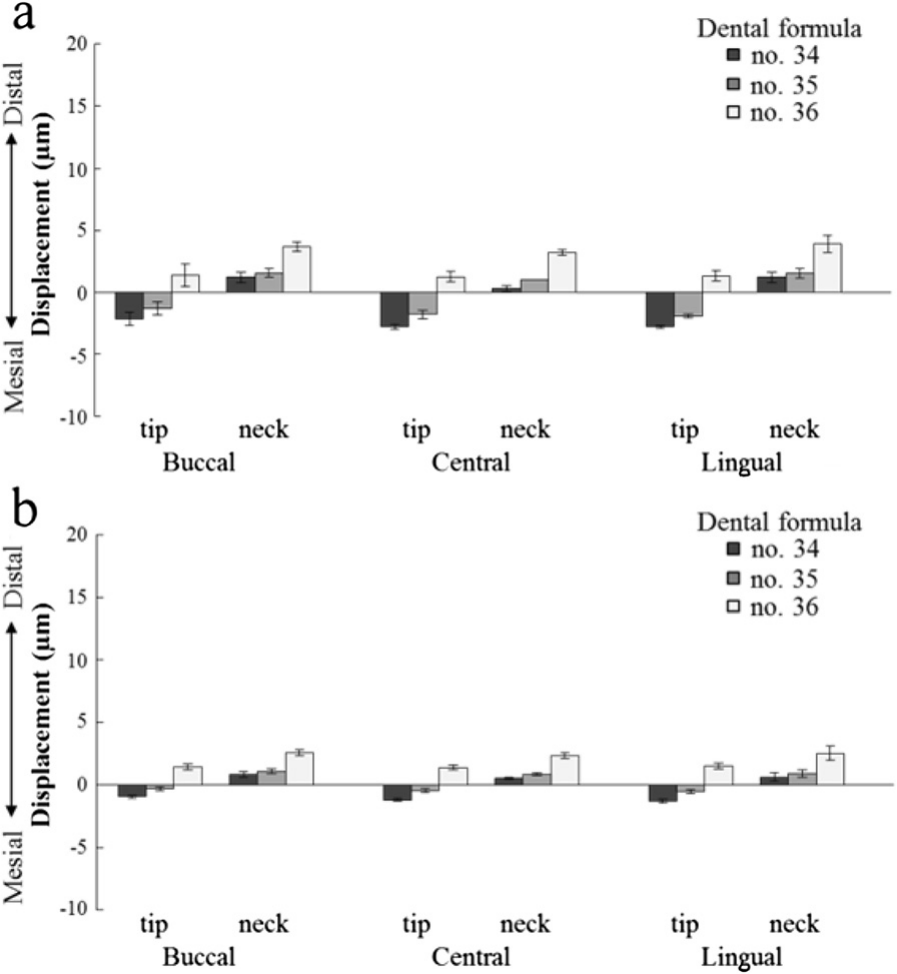
Displacement in the mesiodistal direction (*y*-axis). **(a)** The contact model and **(b)** the fixation model.

At all three loading points, no. 34 and no. 35 showed displacements that were rotated and inclined towards the distal direction; in contrast, no. 36 showed a displacement that moved parallel to the distal direction (Figure 9). Compared with the contact model, the fixation model had less displacement, but aspects of the displacements showed similar tendencies. The results of the ANOVA showed that significant factors for three-dimensional displacement were assessment site, dental formula, and loading point in the contact model, and assessment site and dental formula in the fixation model (*p* < 0.05) (Table 4).

**Table 4:** **Three-way ANOVA (displacement in the mesiodistal direction [** ***y*** **-axis])**

**Source**	**Sum of squares**	***df***	**Mean squared**	***F*** **value**	***p*** **value**
Contact model					
A: Observed area	116.630	1	116.630	197.889	0.005**
B: Dental formula	109.456	2	54.728	171.628	0.000**
C: Loading points	2.544	2	1.272	10.139	0.027*
Fixation model					
A: Observed area	26.825	1	26.825	695.121	0.001**
B: Dental formula	48.534	2	24.267	323.554	0.000**
C: Loading points	0.406	2	0.203	1.945	0.257

*p < 0.05, **p < 0.01.

#### Three-dimensional displacement in the inferior-superior direction (*z*-axis)

**Figure 10:**
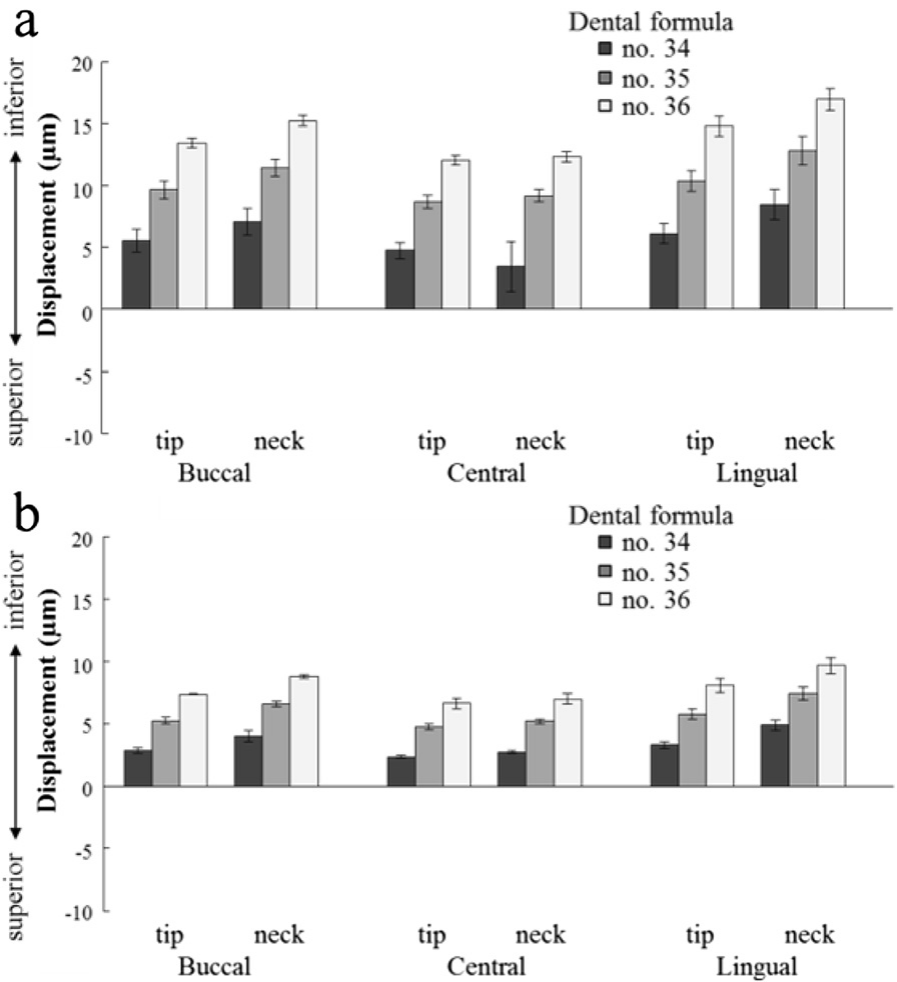
Displacement in the inferior-superior direction (*z*-axis). **(a)** The contact model and **(b)** the fixation model.

At all three loading sites, no. 36 had the greatest displacement; the more mesial the implant, the less the displacement, and the distal portions showed a sinking displacement (Figure 10). Central loading resulted in the least displacement; buccal and lingual loading showed substantially similar displacements. Compared with the contact model, the fixation model demonstrated less displacement, but aspects of the displacements showed similar tendencies. The results of the ANOVA showed that significant factors for the three-dimensional displacement were assessment site, dental formula, and loading point in both the contact and fixation models (*p* < 0.05) (Table 5).

**Table 5:** **Three-way ANOVA (displacement in the inferior-superior direction [** ***z*** **-axis])**

**Source**	**Sum of squares**	***df***	**Mean squared**	***F*** **value**	***p*** **value**
Contact model					
A: Observed area	22.324	1	22.324	68.424	0.014*
B: Dental formula	610.338	2	305.169	915.448	0.000**
C: Loading points	92.755	2	46.377	22.619	0.007**
Fixation model					
A: Observed area	16.600	1	16.600	360.045	0.003**
B: Dental formula	190.012	2	95.006	2641.293	0.000**
C: Loading points	27.806	2	13.903	78.581	0.001**

*p < 0.05, **p < 0.01.

### Equivalent stress in the FEA models

#### Stress distribution

**Figure 11:**
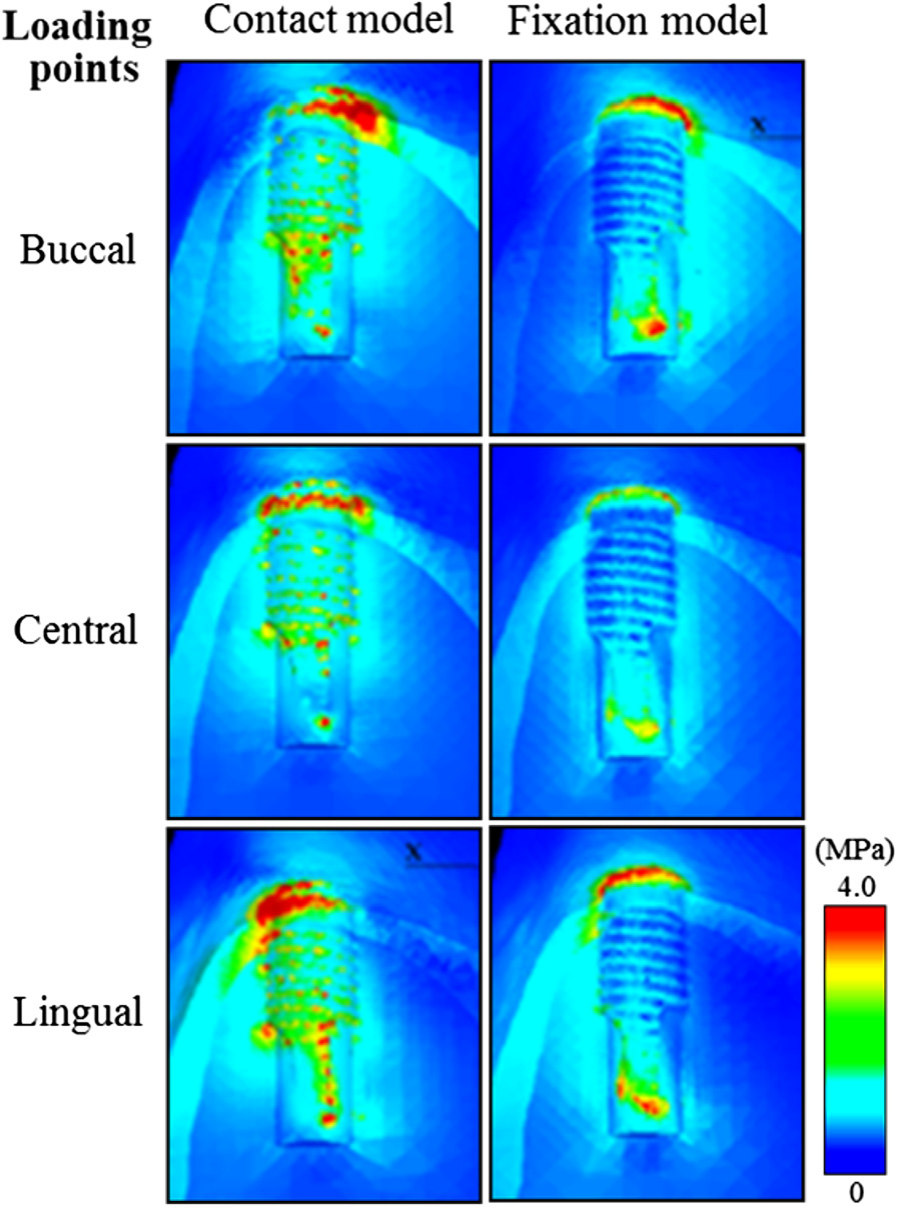
The distribution of equivalent stress (MPa) around the first molar.

Figure 11 shows the equivalent stress distribution for each loading point in the first molar implant section under 100 N of vertical loading. The concentrated site of equivalent stress generated in the artificial mandibular bone in the contact and fixation models was on the buccal side of the bone surrounding the implant neck during buccal loading, the lingual side during lingual loading, and the distal center during central loading. This means that a stress concentration was observed in the bone surrounding the implant neck on the loading side. A minute amount of stress generation was observed at the implant tip and threads as well. The contact model had a larger stress concentration range than the fixation model.

#### Equivalent stress values

**Figure 12:**
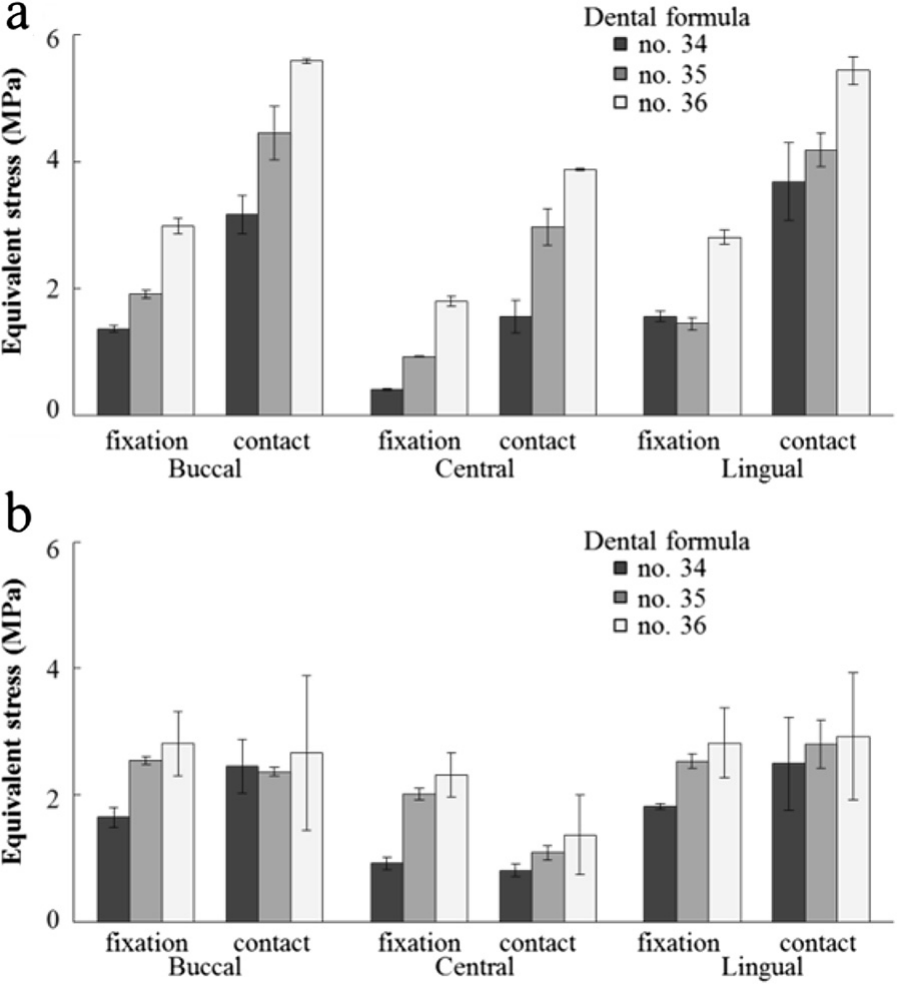
Equivalent stresses at **(a)** the neck and **(b)** the tip of the implant.

Figure 12, Table 6, and Table 7 show the results for the equivalent stress values of the implants at each loading point under 100 N of vertical loading.

**Table 6:** **Three-way ANOVA (equivalent stress)**

**Source**	**Sum of squares**	***df***	**Mean squared**	***F*** **value**	***p*** **value**
The neck of the implant					
A: Boundary conditions	64.725	1	64.725	230.721	0.004**
B: Dental formula	29.391	2	14.695	365.583	0.000**
C: Loading points	20.123	2	10.062	140.179	0.000**
The tip of the implant					
A: Boundary conditions	0.037	1	0.037	0.044	0.854
B: Dental formula	5.941	2	2.971	2.684	0.182
C: Loading points	14.050	2	7.025	39.959	0.002**

**p < 0.01.

**Table 7:** **Coefficients of variation for equivalent stresses**

**Model**	**Loading points**			
	**Buccal loading**	**Central loading**	**Lingual loading**	**Average**
The neck of the implant				
Contact model				
No. 34	9.62	16.43	16.75	14.27
No. 35	9.39	9.81	6.43	8.54
No. 36	0.72	0.52	4.04	1.76
Fixation model				
No. 34	3.36	3.24	5.69	4.10
No. 35	3.04	0.54	7.03	3.53
No. 36	4.03	4.44	3.91	4.13
The tip of the implant				
Contact model				
No. 34	17.31	11.83	29.53	19.55
No. 35	2.83	10.34	13.62	8.93
No. 36	45.69	45.99	34.13	41.94
Fixation model				
No. 34	9.67	10.44	2.22	7.45
No. 35	2.33	4.53	4.43	3.76
No. 36	18.09	15.09	19.79	17.65

Coefficient of variation (%) = (S.D.)/(mean).

##### Equivalent stress values in the bone surrounding the implant neck

The equivalent stress values of the fixation model were lower at all loading sites than the contact model (Figure 12a). The value was smallest under central loading; buccal loading and lingual loading showed equivalent values. For more distal implants, greater stress values were exhibited. The results of the ANOVA showed that in bone surrounding the implant neck, significant factors for the equivalent stress value were boundary conditions, dental formula, and loading point (*p* < 0.01) (Table 6). The maximum CV was 16.75% in the contact model and 7.03% in the fixation model (Table 7).

##### Equivalent stress values in the bone surrounding the implant tip

Central loading resulted in the lowest equivalent stress value, while buccal and lingual loading showed substantially similar values (Figure 12b). In the bone surrounding the implant tip, the loading point was a significant factor for the equivalent stress value (*p* < 0.01) (Table 6). The maximum CV was 45.99% in the contact model and 19.79% in the fixation model (Table 7).

## Discussion

### Experimental methods

#### Experimental model

When a three-dimensional FEA is used to analyze the mechanics of peri-implant bone, it is ideal to construct an FEA model that approximates the material properties and structures of an actual mandible. Moreover, the results should be compared with the behavior of an implant in an actual mandible. However, in an actual oral cavity, individual differences exist resulting from bone morphology and physical properties; therefore, it is difficult to conduct experiments under constant conditions and to obtain results that can be applied to all individuals. In other words, to systematically analyze the mechanics of peri-implant bone, an artificial bone model in which individual differences can be eliminated is regarded as valid. The artificial mandibular bone used in this study was regarded as type II in the Lekholm and Zarb classification [18] and had been fabricated on the assumptions of having adequate bone quality, internal structure, and morphology for clinically valid implant therapy. It is difficult to say whether the experimental model was an ideal model because the experimental model has different material properties from those of an actual mandible. However, the purpose of this study was not to compare it with the behavior of an implant in an actual oral cavity; the purpose was to perform a comparison between an experimental model and FEA model to verify the reproducibility and validity of a three-dimensional finite element model. Therefore, we used artificial mandibular bone in the experimental model rather than an actual mandible.

#### FEA models

In many reports on the three-dimensional FEA of implants, loading was carried out using a simplified FEA model in which the cancellous bone interior was regarded as a homogeneous body [19-23]. This is partly because X-ray CT imaging does not provide adequate resolution, and it is difficult for CT to accurately reflect the state of contact between the trabecular structure and the implant [24]. Therefore, it is inevitable that loading will be performed using a simplified FEA model in which the cancellous bone interior is regarded as being a homogeneous body, as was the case in the present experiment. FEA in industry has been utilized as a ‘rough analysis’ (first-order analysis) tool by simplifying the details in order to ascertain an overall tendency in the first stages of structural design [25]. Therefore, in the present study, a ‘first-order analysis’ was utilized to ascertain the behavioral tendencies of the implants as a first step before proceeding with an analysis of implant mechanics using a three-dimensional FEA. As has been performed in many reports on the FEA of implants, we verified the validity of the FEA models by studying the extent to which the actual behaviors were reproduced when the trabecular structure in the FEA models was simplified and compared with the experimental model.

### Experimental results

#### Implant displacement under loading conditions

In the experimental model, an implant cavity 3.0 mm in diameter was formed prior to embedding an implant 3.75 mm in diameter. In theory, the threads were completely mechanically fitted to the artificial mandibular bone. It does not osseointegrate, but does represent the circumstances of immediate loading in a state of full contact with the bone. The contact model reproduced the state of contact between the bone and implant in the experimental model; theoretically, displacements under loading conditions should show values equivalent to those in the experimental model. Nonetheless, the displacement under loading conditions in the contact model had values 1/3 to 1/4 of those observed in the experimental model. There are three conceivable possibilities. One is that it is possible that consecutive loading of the superstructure on the buccal and lingual sides causes implant loosening. In such a case, displacement under loading conditions would be larger than in the experimental model. Sato et al. [26] reported that the yield tensile load of a screw was 656 N. The fatigue limit causing screw loosening or fracture is half of the yield tensile load, or 328 N. Using a geometric analysis, the largest tensile force in the gold screw after a buccal loading of 100 N was 73 N. In this reported case, if more than 450 N was applied to the loading point, the screw will loosen or fracture. Therefore, 100 N of consecutive superstructure loading on the buccal and lingual sides was not a cause of implant loosening. Furthermore, it is not a reason for displacement under loading conditions in the experimental model to be greater. The second reason is that the experimental model was simply placed on the worktable. Therefore, there is the possibility that minute movements occurred during the vertical loading process. Additionally, measured implant displacement under loading may have overestimated the actual displacement. In order to reproduce complete constraint conditions in FEA models under loading conditions, the bottom surface of the artificial mandibular bone should be adhered completely to the worktable with adhesive; this should stabilize the experimental model and minimize minute movements during the loading process. The third reason is that it is thought that the Young’s modulus assigned to the FEA models was different from the actual Young’s modulus of artificial mandibular bone. In an FEA, the physical properties assigned to elements reportedly have a major impact on the analysis results [27,28]. Nomura et al. [29] reported that displacement under loading conditions increases when the Young’s moduli of the cortical and cancellous bone are reduced. We used the manufacturer’s publicly disclosed values for the Young’s modulus of the artificial mandibular bone, but it is not clear how these values were measured. In particular, with respect to artificial cancellous bone, the interior has become a foam state and it is thought that the Young’s modulus is smaller than the publicly disclosed value. In such a case, displacement under loading conditions would be greater than measured in this study’s FEA models and would be nearer to the displacement under loading conditions in the experimental model. An accurate method for measuring the Young’s modulus also requires future study.

In the experimental and contact models, the absolute values of displacement under loading were different, but aspects of the displacement under loading conditions caused by differences in the loading points were similar and showed similar tendencies. The correlation coefficient of the two was 0.925, representing a significant and strong correlation (*p* < 0.01). This shows that the behavioral tendencies of the contact model are reflective of those in the experimental model and that the results obtained had high validity. The CV of the displacement under loading conditions was calculated as about 10% in some areas of the FEA models, but the mean was about 5% for all three models, representing a relatively low value. This suggests that all models had highly reproducible displacements under loading and that the results obtained had high validity. Although there are limitations to the reproducible range, it appears possible to infer phenomena to an extent if the properties are understood and the limitations are known. Analysis by three-dimensional finite element models has been shown to be an effective means for studying the behavioral tendencies of implants under loading conditions.

#### Three-dimensional displacement in the FEA models

##### Directions of implant displacement

Hotta et al. [30] measured the amount of displacement under the loading of implants placed in human mandibles. When a load is applied at a location that deviates from the long-axial direction of implant more buccally and lingually, the forces from rotation and inclination are propagated to the implant as an eccentric axial load. The implant displacement of eccentric axial loading has been reported to be larger than that during long-axial direction loading. Awazawa et al. [31] measured displacements under the loading of implants placed in canine mandibles and reported no substantial difference in displacement based on whether the loading direction was towards the buccal or lingual side during buccolingual loading. These reports are consistent with the results of *x*-axis displacement in this study and support the clinical validity of the constructed FEA models.

##### Impacts of different boundary conditions on displacement

It has been reported that when micromovement of an implant occurs, an ingrowth of soft tissue occurs after the implant is embedded; therefore, it is difficult to achieve osseointegration [32-34]. Brunski et al. [35] reported that when immediate loading or early loading is carried out, micromovements of the implant should be controlled to 100 μm or less and excessive movement of the implant not only impairs osseointegration but also encourages the growth of connective tissue. In the experimental results of the present study, the displacement of the contact model, which assumed immediate loading, showed greater values than the fixation model, which assumed delayed loading. That is to say, in the FEA models constructed in the present study, the results support the notion that micromovements are likely to occur during immediate loading and that suppressing these as much as possible is necessary for successful osseointegration.

#### Equivalent stress values and their occurrence sites

When assessing stress values of FEA models, it is desirable to do so after confirmation of the validity of the models [11]. Therefore, we first confirmed the validity of the FEA models from comparisons of the correlation coefficients of the displacements under loading conditions in the experimental and contact models. Then, the equivalent stress values and their sites of occurrence were assessed to examine how peri-implant bone is impacted by differences in boundary conditions and loading points.

##### Impacts from differences in loading sites

The equivalent stress values in both the contact and fixation models were smallest for central loading, while buccal and lingual loading showed substantially equivalent values greater than that of central loading. Equivalent stress occurrence sites were observed to be high in bone surrounding the implant neck on the loading side, similar to previous reports [36,37]. Hobo et al. [38] stated that while implants were resistant to vertical pressure, horizontal pressure (bending movements) generated torque in the implants and had more harmful effects; therefore, it would be wise to limit the occlusal contact of the superstructure to vertical pressure and avoid horizontal pressure as much as possible. This was also consistent with reports that lateral loading generated more stress than vertical loading, as is also found in Sato et al.’s report using a geometric analysis [39], and supports the existing clinical concept that a lateral force applied to an implant greatly increases the stress in the surrounding bone.

##### Impacts of differences in boundary conditions

The equivalent stress values of the contact model were higher at the implant neck than the tip, and the stress generation range was also broader. However, in the fixation model, the implant neck and tip had substantially equivalent values and the stress generation range was also narrower than that of the contact model. This shows that under immediate loading conditions, there is a high likelihood that loading applied to the superstructure is also supported by cancellous bone at the implant threads and tip, but the majority is supported by cortical bone at the implant neck. That is, under immediate loading conditions, it is believed there is a need to be mindful of the stress concentration at the implant neck. The material properties of the FEA models constructed in the present study cannot not be compared to actual oral cavity stress values because they differ significantly from an actual bone. However, when regarding how peri-implant bone is impacted by contact between the implant and bone, it is considered sufficiently useful in predicting tendencies.

##### Reproducibility of the equivalent stress values in the FEA models

The CV of the equivalent stress values was calculated to assess the reproducibility of the contact and fixation models. The CV ranged from 0.52 to 45.99, showing a tendency for higher overall values compared with the CV for displacement under loading. The reproducibility of the equivalent stress values had considerable variance from model to model in some regions. In particular, the contact model showed a higher CV at both the neck and tip. In an analysis of contact conditions, moving the nodes also dramatically changed the stress and strain occurring at the interface; a stress concentration was also generated depending on the shape of the model [40]. Though the FEA models were fabricated under similar conditions, it appears that a minute error in shape caused in the element divisions appeared in the form of a large error in equivalent stress values. It is necessary to verify whether the numerical stress values obtained from the FEA have validity by attaching a strain gauge to an experimental model and observing correlations in stress values between the experimental and FEA models.

## Conclusions

With the objective of verifying the reproducibility and validity of three-dimensional finite element models, we fabricated finite element models and multiple models in which implants were embedded in artificial mandibles and compared implant displacements under various loading conditions; the results obtained produced the following conclusions:

1. The CVs as calculated from the amount of displacement under loading in the experimental, contact, and fixation models were about 5% to 10%, and all models had high reproducibility with respect to implant displacement under loading.
2. If three loading points were applied in the experimental and FEA models, the aspects of vertical implant displacement were similar in both.
3. The correlation coefficient of implant displacement under loading conditions in the experimental and contact models was 0.925, representing a high correlation, and the validity of the displacement under loading was high in the contact model.
4. The CV as calculated from equivalent stress values in the contact and fixation models ranged from 0.52% to 45.99%, and the reproducibility of the equivalent stress values showed considerable variance from model to model.

The above results show that the three-dimensional finite element models were reflective of displacement tendencies in the experimental model, and the results obtained had high reproducibility and validity. However, it was shown that when the validity of the absolute value of displacement was low, the reproducibility of the equivalent stress values was also inferior. Three-dimensional FEA was observed to be an effective means for investigating the behavioral tendencies of implants under loading conditions. Although there are limitations to the reproducible range, it is possible to infer phenomena to an extent if the properties are understood and the limitations are known. However, the results need to be interpreted cautiously, with a full understanding that FEA methods are purely numerical data that are mathematically enumerated.

## Abbreviations

FEA: finite element analysis
CT: computed tomography
CV: coefficient of variation
CAD/CAM: computer-aided design/computer-aided manufacturing
ANOVA: analysis of variance
